# Identification of UBE2I as a Novel Biomarker in ccRCC Based on a Large-Scale CRISPR-Cas9 Screening Database and Immunohistochemistry

**DOI:** 10.3389/fmolb.2022.813428

**Published:** 2022-02-08

**Authors:** Feng Li, Li Lai, Zhijie You, Hui Cheng, Guodong Guo, Chenchen Tang, Luyun Xu, Hongxia Liu, Wenting Zhong, Youyu Lin, Qingshui Wang, Yao Lin, Yongbao Wei

**Affiliations:** ^1^ Shengli Clinical Medical College, Fujian Medical University, Fuzhou, China; ^2^ Department of Pathology, Fujian Provincial Hospital, Fuzhou, China; ^3^ The School of Basic Medical Sciences, Fujian Medical University, Fuzhou, China; ^4^ Central Laboratory, Fujian Provincial Hospital, Fuzhou, China; ^5^ Key Laboratory of Optoelectronic Science and Technology for Medicine of Ministry of Education, College of Life Sciences, Fujian Normal University, Fuzhou, China; ^6^ Fujian Provincial Key Laboratory of Hepatic Drug Research, Fuzhou, China; ^7^ Central Laboratory at the Second Affiliated Hospital of Fujian Traditional Chinese Medical University, Innovation and Transformation Center, Fujian University of Traditional Chinese Medicine, Fuzhou, China; ^8^ Department of Urology, Fujian Provincial Hospital, Fuzhou, China

**Keywords:** CRISPR-cas9 screening, ccRCC, cell cycle, UBE2I, nuclear translocation

## Abstract

**Background:** The genome-wide CRISPR-cas9 dropout screening has emerged as an outstanding approach for characterization of driver genes of tumor growth. The present study aims to investigate core genes related to clear cell renal cell carcinoma (ccRCC) cell viability by analyzing the CRISPR-cas9 screening database DepMap, which may provide a novel target in ccRCC therapy.

**Methods:** Candidate genes related to ccRCC cell viability by CRISPR-cas9 screening from DepMap and genes differentially expressed between ccRCC tissues and normal tissues from TCGA were overlapped. Weighted gene coexpression network analysis, pathway enrichment analysis, and protein–protein interaction network analysis were applied for the overlapped genes. The least absolute shrinkage and selection operator (LASSO) regression was used to construct a signature to predict the overall survival (OS) of ccRCC patients and validated in the International Cancer Genome Consortium (ICGC) and E-MTAB-1980 database. Core protein expression was determined using immunohistochemistry in 40 cases of ccRCC patients.

**Results:** A total of 485 essential genes in the DepMap database were identified and overlapped with differentially expressed genes in the TCGA database, which were enriched in the cell cycle pathway. A total of four genes, including UBE2I, NCAPG, NUP93, and TOP2A, were included in the gene signature based on LASSO regression. The high-risk score of ccRCC patients showed worse OS compared with these low-risk patients in the ICGC and E-MTAB-1980 validation cohort. UBE2I was screened out as a key gene. The immunohistochemistry indicated UBE2I protein was highly expressed in ccRCC tissues, and a high-level nuclear translocation of UBE2I occurs in ccRCC. Based on the area under the curve (AUC) values, nuclear UBE2I had the best diagnostic power (AUC = 1). Meanwhile, the knockdown of UBE2I can inhibit the proliferation of ccRCC cells.

**Conclusion:** UBE2I, identified by CRISPR-cas9 screening, was a core gene-regulating ccRCC cell viability, which accumulated in the nucleus and acted as a potential novel promising diagnostic biomarker for ccRCC patients. Blocking the nuclear translocation of UBE2I may have potential therapeutic value with ccRCC patients.

## Introduction

Kidney cancer is a common urinary system disease with a high incidence, and approximately 90% of it is renal cell carcinoma (RCC) (Siegel et al., 20172017; Chen et al., 2019). Clear cell renal cell carcinoma (ccRCC) is a highly aggressive renal malignant tumor, accounting for nearly 80% of RCCs (Mekhail et al., 2005; Cairns, 2010). The existing traditional treatment methods, such as surgical resection, chemotherapy, and radiotherapy, seem to be ineffective against this aggressive tumor (Rini et al., 2009; Ghatalia et al., 2018). Despite the continuous advancement of cancer treatment, the mortality rate of ccRCC is still rising. Sixty percent of ccRCC patients died within the first 3 years, and 30% of ccRCC patients were diagnosed with metastases (Mendoza-Alvarez et al., 2019; Zhou et al., 2020). Therefore, it is of great significance to determine the effective biomarkers responsible for the development of ccRCC.

Recently, CRISPR-cas9 screening is becoming a powerful tool for precision medicine (Doudna and Charpentier, 2014; Kurata et al., 2018; Sun et al., 2021). Combining cas9 with guide RNA libraries can help screen genes that contribute to specific biological phenotypes or diseases in a high-throughput manner (Joung et al., 2017). Project DepMap uses CRISPR-Cas9 tools to knock out each gene individually to identify candidate genes that are critical to tumor survival or proliferation. CERES algorithm was developed to calculate gene-knockout effects, and CERES scores of 0 and −1 represent the median effects of nonessential genes and common core essential genes per cell line, respectively (Meyers et al., 2017). In a tumor, the CERES score may depend on the genotype, transcriptome, and the lineage of cell lines (Shimada et al., 2021). In principle, genes that are essential only in a few cell lines may become better drug targets because inhibiting their function is unlikely to cause toxicity in noncancerous tissues. For example, some certain tumor cells strongly require epidermal growth factor receptor, but normal bone marrow stem cells do not, making it a potentially good target (Wang et al., 2006).

In the present study, we aim to identify candidate genes that are differentially expressed in ccRCC tissues and contribute to the viability of ccRCC cells. Then, a module partition analysis was performed with weighted gene coexpression network analysis (WGCNA) by using these candidate genes, followed by functional enrichment analyses. Then, a predictive model with prognostic significance was constructed and verified. Finally, the expression of screened core genes was verified by immunohistochemistry (IHC).

## Methods

### Identifying Essential ccRCC Genes

The Cancer Dependency Map (https://depmap.org/portal/) is a website based on a large-scale multiomics screening project (Meyers et al., 2017; Shi et al., 2021). The CERES algorithm was used to calculate dependency scores for approximately 17,000 candidate genes to identify genes that are critical to proliferation and survival. A positive score of CERES indicates that knocking out the gene promotes the survival and proliferation of the cell line, whereas a negative score indicates that knocking out the gene inhibits survival and proliferation. Candidate genes are defined as essential genes with a CERES score <−1 in 75% of ccRCC cell lines (Ho et al., 2021).

### Clinical Data Acquisition and Extraction

Transcriptome RNA-sequencing data and clinical information of ccRCC were downloaded from the TCGA data portal (https://cancergenome.nih.gov/). There were 538 cases of ccRCC tissues and 72 cases of normal tissues. The gene expression data of ccRCC used for validation cohort were obtained from ArrayExpress (https://www.ebi.ac.uk/arrayexpress) (Athar et al., 2019) and the International Cancer Genome Consortium (ICGC) (https://icgc.org/) (Zhang et al., 2019a). The accession number of ArrayExpress is E-MTAB-1980, including 106 who had follow-up information. The accession number of ICGC is RECA-EU, including 91 who had follow-up information.

### Differentially Expressed Genes

R Limma package was used to calculate differentially expressed genes (DEGs) between ccRCC tissues and normal kidney patients in the TCGA database. Genes with *p* < 0.05 and fold change >1.5 were defined as DEGs.

### Functional Enrichment Databases

GO analysis of candidate genes was performed using the Metascape web tool (www.metascape.org) (Zhou et al., 2019). The analysis of Metascape was performed with default settings. We chose Metascape instead of DAVID, because Metascape’s database is renewed monthly to ensure the content remains current.

### WGCNA Analysis

In the study, WGCNA was performed with the WGCNA R package (Langfelder and Horvath, 2008). A power of *β* = 5 and a scale-free *R* ^2^ = 0.95 were selected as soft-threshold parameters to ensure a signed scale-free coexpression gene network. A cluster dendrogram was created based on the topological overlap matrix with a minimum cluster size of 20. In total, six modules were generated.

### Gene Signature Construction

The least absolute shrinkage and selection operator (LASSO) was used to construct the 12-cell-cycle–related gene signature by TCGA ccRCC transcriptome data. L1-norm was applied to penalize the weight of the features. A cell-cycle–related gene signature–based risk score formula was established by including individual normalized gene expression values weighted by their LASSO Cox coefficients: risk score = (UBE2I × 0.029 + NCAPG × 0.212 + NUP93 × 0.029 + TOP2A × 0.012).

### Estimation of the Tumor Microenvironment

The CIBERSORT algorithm and ESTIMATE algorithm in the CIBERSORT and estimate package of the R software was used, respectively. We used the CIBERSORT algorithm to estimate data on tumor-infiltrating immune cells. CIBERSORT can transform the gene expression matrix into the corresponding infiltrating immune cell expression matrix. ESTIMATE algorithm was used to calculate the estimate scores and immune scores of ccRCC samples.

### Patients With ccRCC Recruitment

Forty ccRCC specimens from the patients of Fujian Provincial Hospital were selected. The study was performed with the approval of the Ethics Committee of Fujian Provincial Hospital and complied with the Helsinki Declaration. The written informed consent was obtained from all participating ccRCC patients.

### Immunohistochemistry Analysis

Here, we measured the UBE21 protein in 40 ccRCC tissues and 40 adjacent normal kidney tissues by IHC. The IHC process was performed as previously described (Li et al., 2018; Wang et al., 2019a; Lin et al., 2021). After deparaffinization, the tissue sections were incubated with anti-UBE2I (ab75854, 1:800 dilution; Abcam) overnight at 4°C. The sections were washed with PBS three times and incubated with horseradish peroxidase–conjugated secondary antibody at room temperature for 30 min. Finally, the sections were counterstained with diaminobenzidine solution and 20% hematoxylin and dehydrated.

### Evaluation of the Immunohistochemical Results

IHC-stained sections for UBE2I protein were reviewed under a microscope and separately evaluated by two independent pathologists using uniform criteria, and nuclear and cytoplasmic staining was scored separately. The immunohistochemical score was calculated by combining the staining intensity score with the proportion score (percentage of positively stained cells) and was described in our previous study (Li et al., 2018).

### Cell Culture and Transfection

The ccRCC A-498 cells and 786-O were obtained from ATCC (American Type Culture Collection, Manassas, VA, USA). A-498 cells and 786-O cells were, respectively, cultured in MEM Alpha medium (Gibco by Life Technologies, Grand Island, NY, USA) and PRMI 1640 (Gibco by Life Technologies) containing 10% fetal bovine serum (BI, Kibbutz Beit Haemek, Israel) and 0.1 mg/mL streptomycin (BBI Life Sciences, Shanghai, China) and 100 U/mL penicillin at 37°C in a humidified incubator with 5% CO_2_. The sequence of shRNA targeting UBE2I was cloned into a pLVX vector. The shRNA sequences were as follows: 5′-CCGG GAA​CTT​CTA​AAT​GAA​CCA​AAT​CTC​GAG​ATT​TGG​TTC​ATT​TAG​AAG​TTC​TTT​TTG-3′. The transfection was performed using Lipofectamine 2000 (Invitrogen, Carlsbad, CA, USA) according to the manufacturer’s guidelines.

### CCK-8 Assay

CCK-8 assay was used to measure cell proliferation. A-498 cells and 786-O cells were seeded onto five 96-well plates (2 × 10^4^ cells/well) in triplicate and cultured for 24, 48, and 72 h. Two hours before absorbance measuring, a CCK-8 solution was added. The absorbance was measured at 450 nm with a microplate reader after incubation at 37°C.

### Statistical Analysis

The statistical correlation was calculated using the *t* test in this study. Overall survival (OS) and disease-free survival were all determined by the Kaplan–Meier method, with the survival curves compared *via* log-rank test. We considered *p* < 0.05 as statistically significant.

## Results

### Identification of Functional Genomic Genes in ccRCC

**FIGURE 1:**
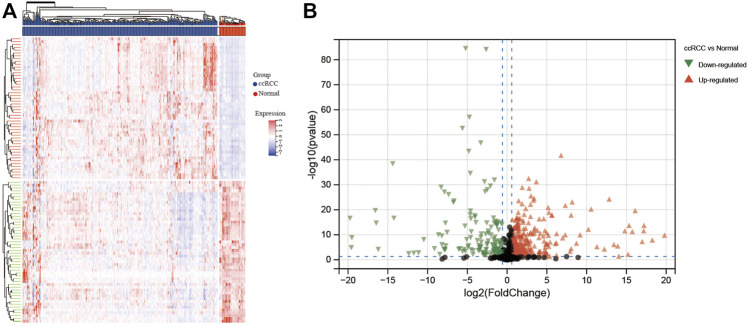
Heatmap and volcano plot for differentially expressed genes identified in ccRCC based on TCGA database. **(A)** Heatmap and **(B)** volcano plot. In the heatmap, blue columns indicate lower gene expression, whereas the red columns indicate higher gene expression. For the volcano plot, black plots represent non-DEGs, green plots represent downregulated DEGs, and red plots represent upregulated DEGs.

CERES dependence score of nine ccRCC cell lines was obtained from the DepMap website. Genes with a CERES score of <−1 in more than 75% of the ccRCC cell lines were defined as candidate genes. A total of 735 genes were marked as candidate genes that are crucial for maintaining survival in nine ccRCC cell lines (Supplementary Figure S1). Next, we attempted to identify aberrantly expressed genes in ccRCC among the 735 candidate genes. Differential gene expression (DEG) analysis revealed that a total of 337 candidate genes were significantly up-regulated and 148 candidate genes significantly down-regulated in ccRCC tissues (n = 538) compared with the normal tissues (n = 72) in the TCGA database (Figure 1).

**FIGURE 2:**
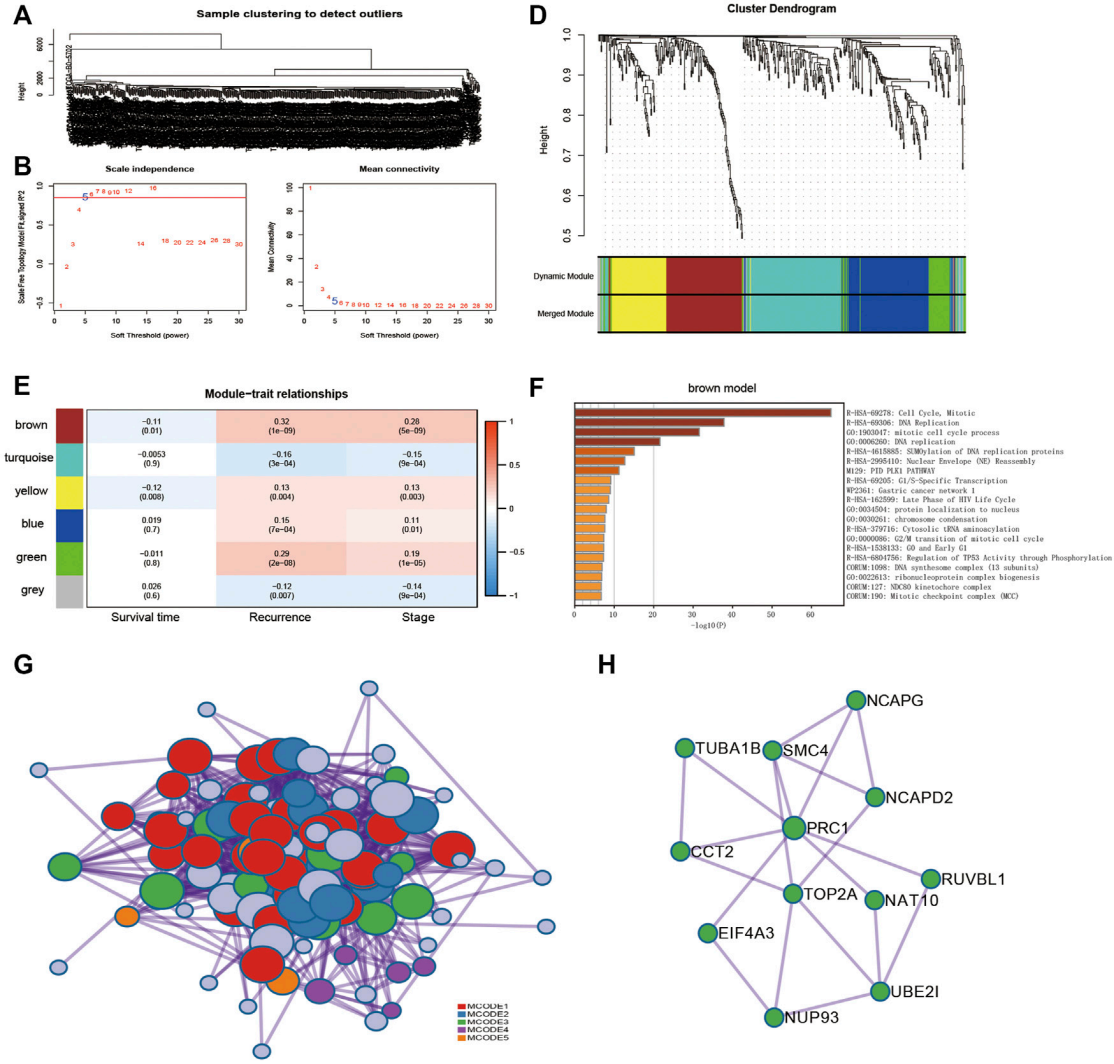
Identification of coexpression module genes using the WGCNA. **(A)** Clustering dendrogram of ccRCC tissues in TCGA database. **(B)** Relationship between scale-free topology model fit and soft thresholds (powers). **(C)** Relationship between the mean connectivity and various soft-thresholds. **(D)** Dendrogram of modules identified by WGCNA. **(E)** Correlation of WGCNA modules with clinical outcomes. **(F)** Functional enrichment for genes within brown modules. **(G)** Protein–protein interaction (PPI) networks for genes within brown modules. **(H)** PPI network of module 3.

WGCNA identifies modules consisting of highly correlated genes. Transcript expression profiles of 485 DEGs for ccRCC tissues (n = 538) in the TCGA database were selected and prepared for WGCNA analysis. We selected the optimal *β* = 5 as the soft thresholding power and the cut height as 0.25 to construct a scale-free network (Figures 2A–C). WGCNA grouped 485 DEGs into six modules based on their coexpression patterns, which includes 135 genes from the turquoise module, 114 genes from the blue module, 104 genes from the brown module, 72 genes from the yellow module, 48 genes from the green module, and 12 genes from the grey module (Figure 2D). Brown module was shown to be negatively associated with tumor recurrence based on the WGCNA results (Figure 2E).

The 104 genes of the brown module were imported into the Metascape tool (https://metascape.org/) for pathway enrichment analysis. The cell cycle pathway was significantly enriched (Figure 2F). Next, the 104 genes were mapped into the protein–protein interaction (PPI) network, of which 102 showed interactions. Five MCODE modules were also identified from the PPI network by the MCODE tool. Functional enrichment of MCODE cluster networks revealed that MCODE 3 (green circles) related to the cell cycle pathway (Figure 2G). There are 12 genes in the MCODE 3, including UBE2I (ubiquitin conjugating enzyme E2 I), NCAPG (non-SMC condensin I complex subunit G), NUP93 (nucleoporin 93), TOP2A (DNA topoisomerase II Alpha), CCT2 (chaperonin containing TCP1 subunit 2), NAT10 (N-acetyltransferase 10), EIF4A3 (eukaryotic translation initiation factor 4A3), RUVBL1 (RuvB-like AAA ATPase 1), SMC4 (structural maintenance of chromosomes 4), NCAPD2 (non-SMC condensin I complex subunit D2), TUBA1B (tubulin alpha 1b), and PRC1 (protein regulator of cytokinesis 1) (Figure 2H).

### Gene Signature Construction and Validation

**FIGURE 3:**
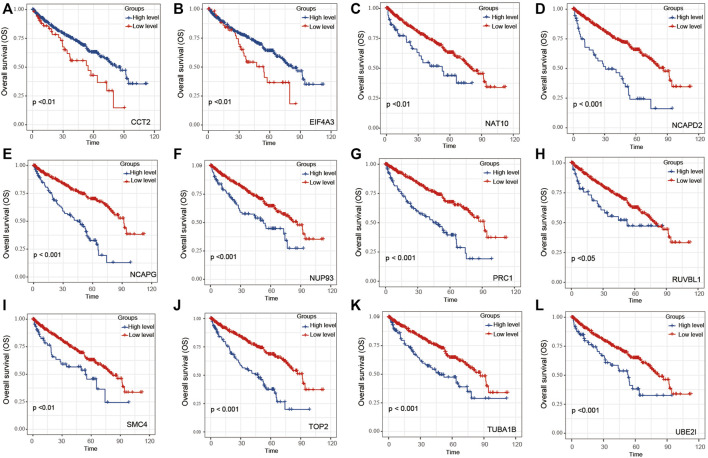
The OS of 12 genes within module 3 for ccRCC patients. Overall analysis for the prognostic value of **(A)** CCT2, **(B)** EIF4A3, **(C)** NAT10, **(D)** NCAPD2, **(E)** NCAPG, **(F)** NUP93, **(G)** PRC1, **(H)** RUVBL1, **(I)** SMC4, **(J)** TOP2, **(K)** TUBA1B, and **(L)** UBE2I expression for OS in ccRCC patients by Kaplan–Meier analysis based on TCGA. The Kaplan–Meier method was used to draw survival curves, and the log-rank test was performed to evaluate survival difference with the best cutoff value.

**FIGURE 4:**
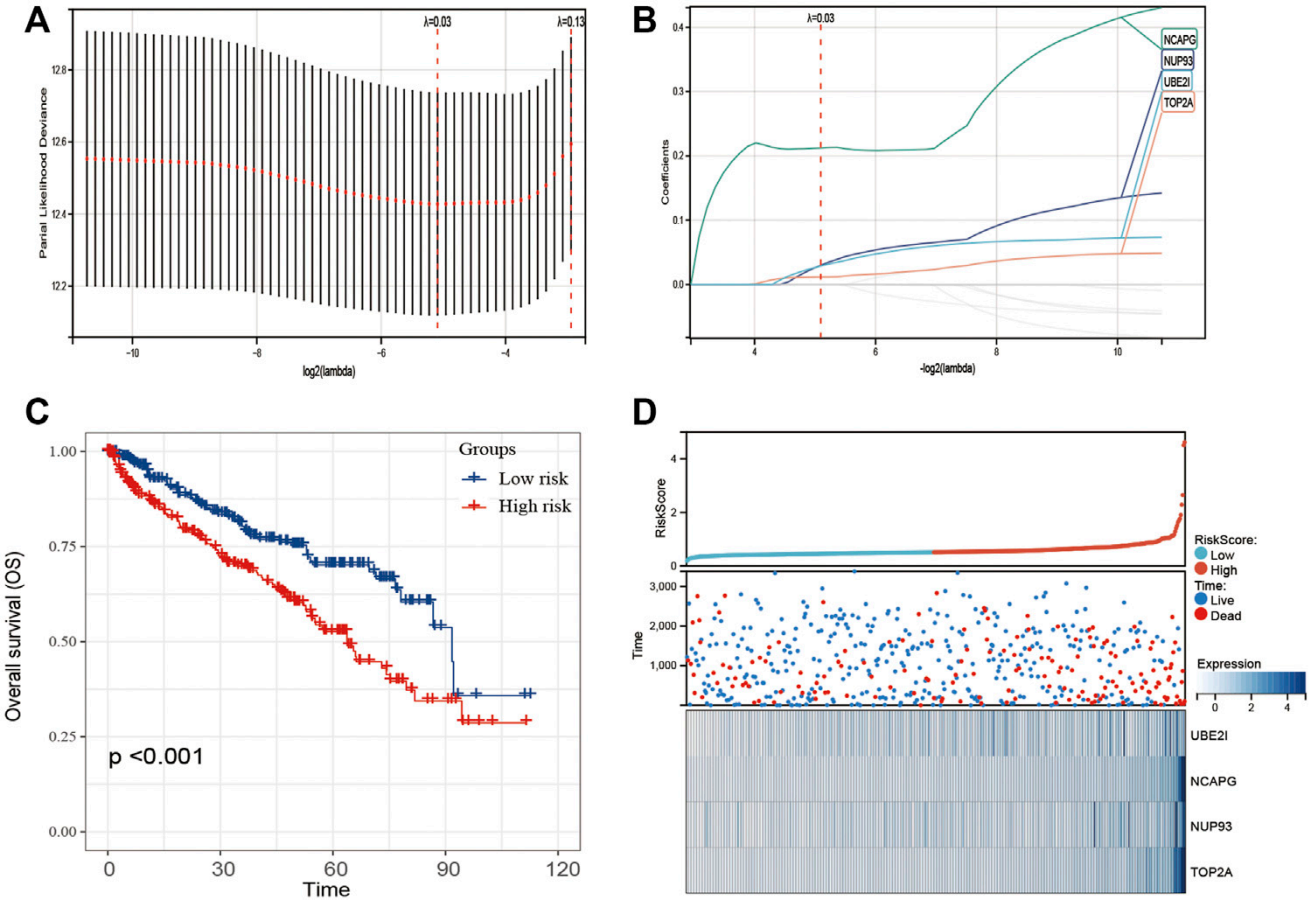
Construction of 12-gene–based classifier to predict prognosis in ccRCC patients. **(A)** Partial likelihood deviance of OS for the LASSO coefficient profiles. **(B)** LASSO coefficient profiles of 12 genes for OS. **(C)** Kaplan–Meier curves to compare overall survival of low-risk and high-risk groups. **(D)** The distribution of risk score, survival status, and mRNA expression levels of ccRCC patients in TCGA cohort.

Univariate Cox regression of 12 genes was performed in the TCGA dataset. The results indicated that CCT2 and EIF4A3 were positively correlated with the prognosis of ccRCC patients (Figures 3A,B); another 10 genes were negatively correlated with the prognosis of ccRCC patients (Figures 3C–L). Later, these 12 genes were included in LASSO analysis with 10-fold cross-validation (Figure 4A). The risk score consisting of four genes that included UBE2I, NCAPG, NUP93, and TOP2A was developed according to the LASSO coefficient and the relative levels of the 12 genes (Figure 4B). The formula of risk score was as follows: risk score = (UBE2I × 0.029 + NCAPG × 0.212 + NUP93 × 0.029 + TOP2A × 0.012).

**FIGURE 5:**
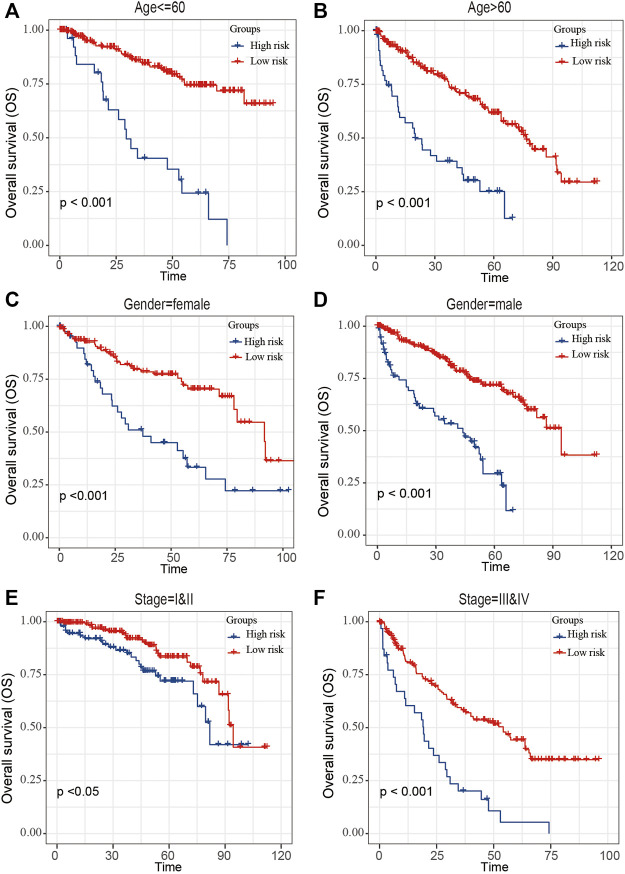
Kaplan–Maier survival curves of overall survival of ccRCC patients according to risk score model in different subgroups. **(A,B)** Prognosis analysis of the subgroups of ccRCC patients with age ≤60 years **(A)** and age >60 years **(B)**. **(C,D)** Prognosis analysis of the subgroups of ccRCC patients with gender = female **(C)** and gender = male **(D)**. **(E,F)** Prognosis analysis of the subgroups of ccRCC patients with stage = I and II **(E)** and stage = III and IV **(F)**. The Kaplan–Meier method was used to draw survival curves, and the log-rank test was performed to evaluate survival differences with the best cutoff value.

The ccRCC patients were divided into high- and low-risk groups based on the risk score. The Kaplan–Meier curve analysis result showed that ccRCC patients with high-risk scores have significantly worse prognosis than those with low-risk scores (Figure 4C). The distribution of risk score, survival status, and mRNA expression levels of ccRCC patients in the TCGA cohort is shown in Figure 4D. Moreover, survival analyses of the patients with low-risk scores and high-risk scores ccRCC based on clinical factors including age, gender, and tumor grade were also performed (Figure 5). These results further confirmed the robust stratification ability of the risk score.

**FIGURE 6:**
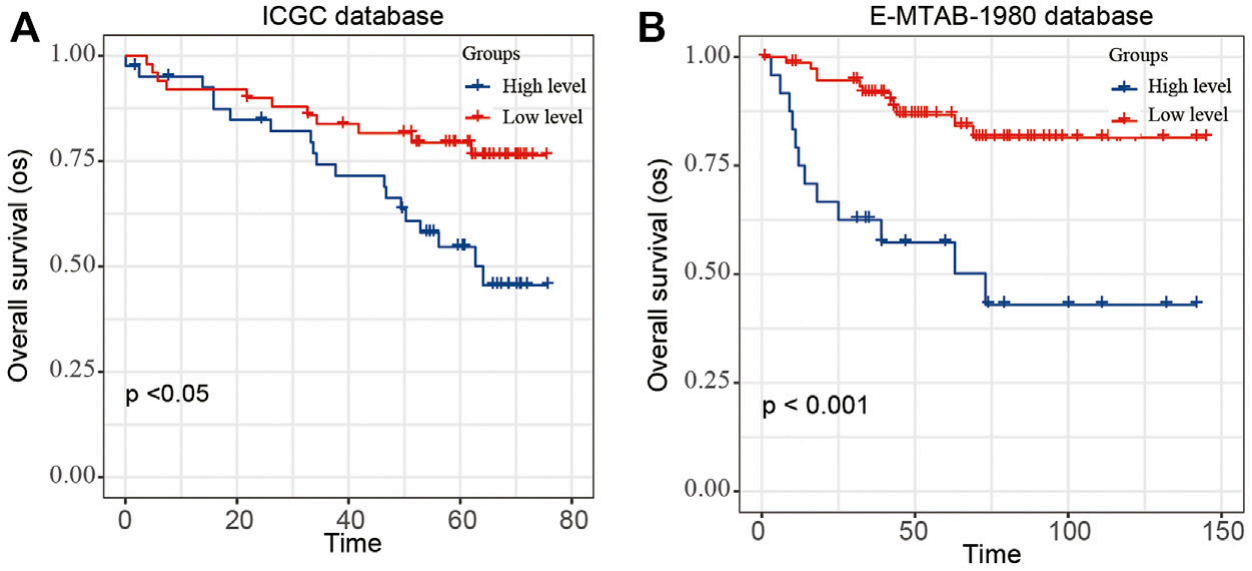
Validation of the risk score model by using E-MTAB-1980 and ICGC database. The Kaplan–Meier plot of the risk score model by using **(A)** ICGC and **(B)** E-MTAB-1980-ccRCC database.

To validate the stability and reliability of the prognostic model, we first downloaded 91 samples with complete clinical information from the ICGC database and 101 samples with complete clinical information from the E-MTAB-1980-ccRCC database as the validation dataset (Figure 6). For each patient, the risk score was calculated using the prognostic model. Patients were divided into the low- and high-risk-score groups, respectively. Kaplan–Meier curve analysis showed that ccRCC patients with high-risk scores had a poor OS than those in the low-risk-score group, indicating good accuracy.

### Tumor Immunity Analysis of Risk Score

**FIGURE 7:**
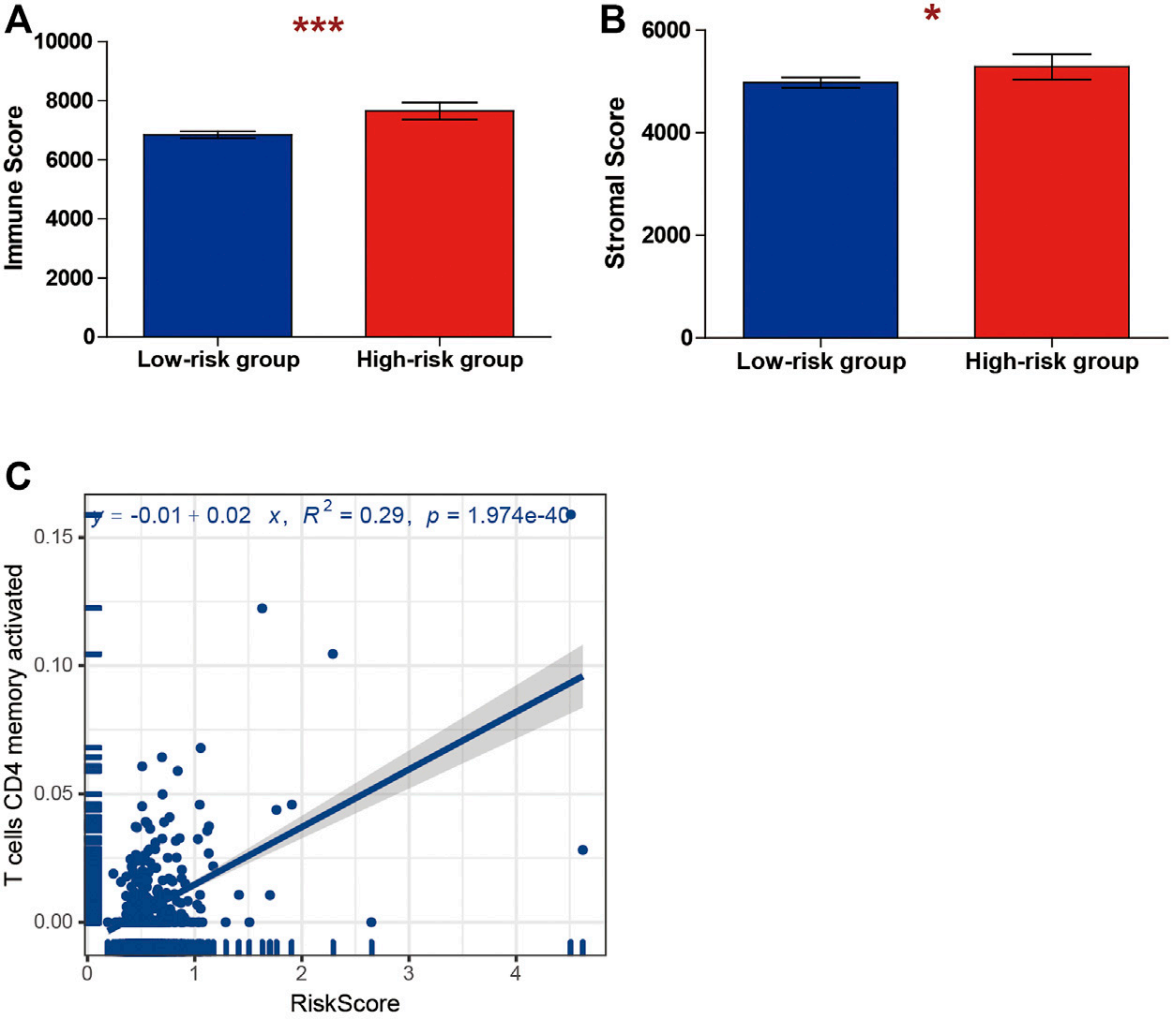
Correlations between risk score and tumor immune microenvironment. The association between the risk score and **(A)** immune and **(B)** stromal score. **(C)** The association between the risk score and T cells CD4 memory activated.

We considered whether the risk score model constituted an important role in the tumor microenvironment and whether it influenced the density levels of tumor-infiltration cells. Through the ESTIMATE algorithm and CIBERSORT algorithm, we estimated the specific fractions of an immune score, estimate score, and 22 immune cells in each sample based on signature expression data from ccRCC patients. The result indicated that the high-risk-score group had a significantly higher stromal score and immune score compared with low-risk-score group (Figures 7A,B). Next, we explored the correlation between immune cell type proportions and the risk score, and the results indicated that only T-cell CD4 memory activated cells were positively correlated to the risk score (Figure 7C).

### Expression of UBE2I in Clinical Specimens

**FIGURE 8:**
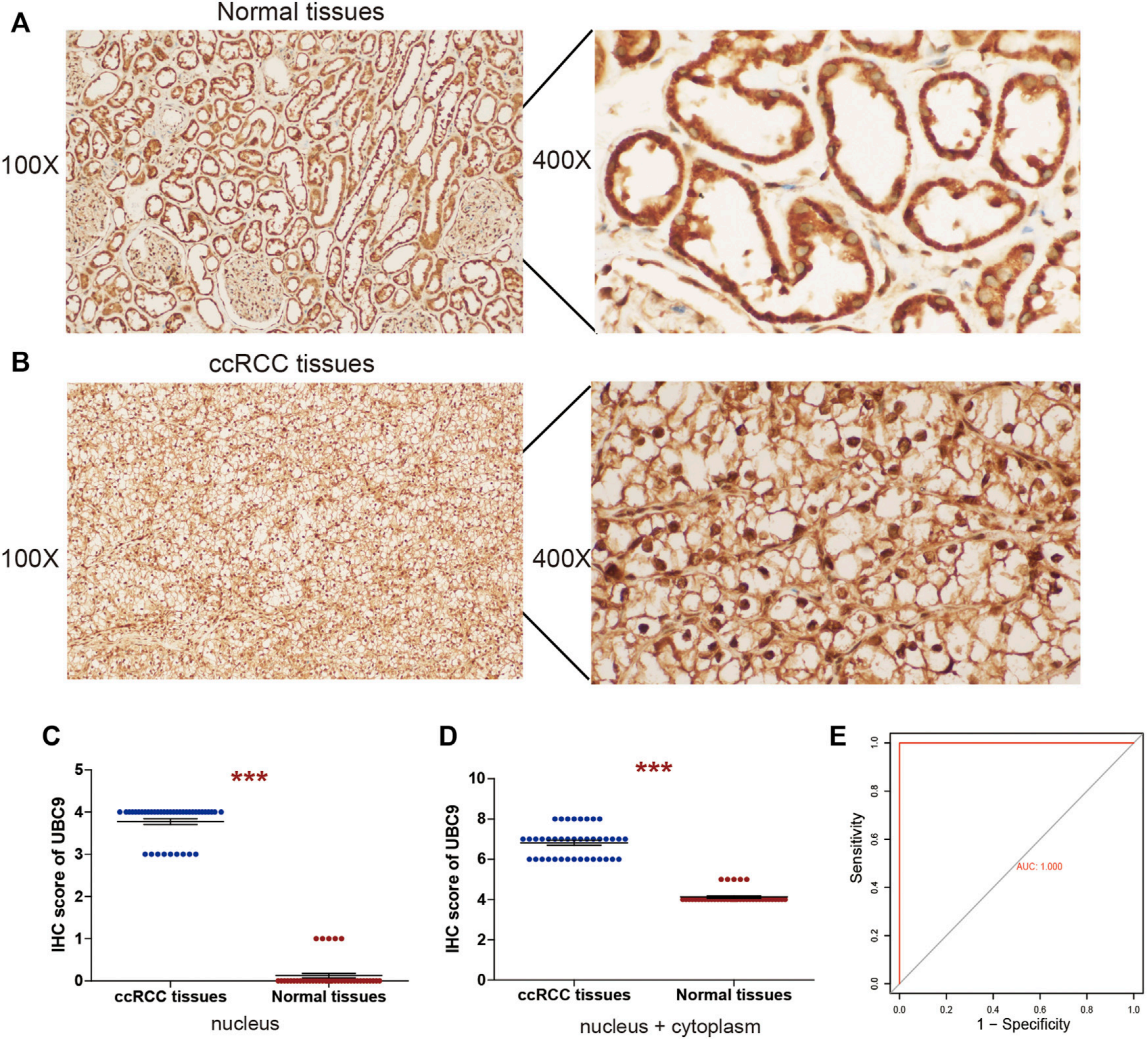
IHC analyses of UBE2I protein expression in ccRCC. Representative IHC images of UBE2I protein in **(A)** normal tissues and **(B)** ccRCC tissues. **(C)** Histogram of IHC score for UBE2I in nucleus. **(D)** Histogram of IHC score for UBE2I in nucleus and cytoplasm. **(E)** Assessment of diagnostic value by ROC curves based on nucleus UBE2I.

Among these four genes, NCAPG, NUP93, and TOP2A, were previously reported to be closely related to kidney disease. However, the role of UBE2I (UBC9 protein-coding gene) in ccRCC has not been explored previously. Then, we collected 40 ccRCC samples and detected the protein expression of UBE2I by IHC; the nucleus and the cytoplasm were scored separately as described in Methods. In normal kidney tissue, UBE2I accumulated in the cytoplasm. However, UBE2I localized to both the cytoplasm and nucleus of ccRCC tissues. Most of the normal kidney tissues did not show UBE2I immunoreactivity in the nucleus (Figure 8A). On the contrary, nuclear UBE2I immunostaining was very high in the ccRCC tissues evaluated (Figure 8B). The total IHC score of UBE2I showed that UBE2I protein was up-regulated in ccRCC compared with normal tissues (Figures 8C,D).

Further on, to assess the capacity of the UBE2I assay to differentiate between ccRCC and normal samples, receiver operating characteristic (ROC) curve analyses were performed, using the IHC data of the UBE2I nucleus as an independent classifier. AUC = 0.5 indicates a random classifier, and AUC = 1 indicates a perfect classifier. Surprisingly, our study achieved a perfect value of 1 (Figure 8E). Subsequently, the association between UBE2I protein and clinicopathological characteristics of patients with ccRCC was assessed. Analysis showed that the UBE2I protein level in the nucleus was associated with tumor size. There was no significant difference between the UBE2I protein level in the nucleus and gender, age, or Fuhrman grade (Table 1).

**TABLE 1:** Characteristics of ccRCC patients and their UBE2I expression level.

Characteristics	Number	Average IHC score in the nucleus	*p* value
Age at diagnosis (years)	—	—	0.2587
≤60	24	3.79	—
>60	16	3.63	—
Gender	—	—	0.4062
Male	26	3.76	—
Female	14	3.64	—
Tumor size	—	—	0.0076
<3.5 cm	23	3.56	—
≥3.5 cm	17	3.94	—
Fuhrman Grade	—	—	0.7794
I + II	24	3.70	—
III + IV	16	3.75	—

### Knockdown of UBE2I Inhibits ccRCC Cell Proliferation

**FIGURE 9:**
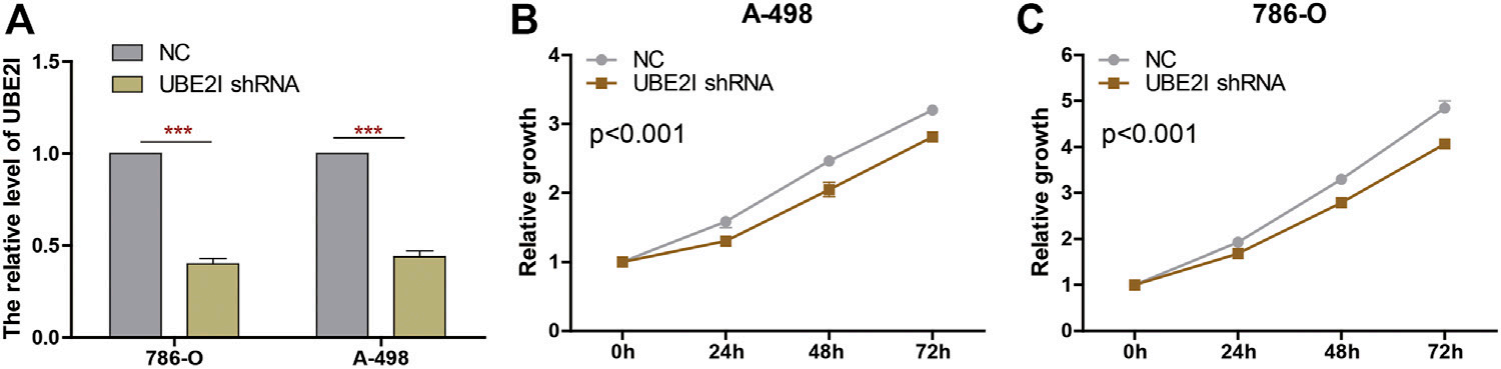
Knockdown of UBE2I inhibits ccRCC cell proliferation. **(A)** The UBE2I expression changes were confirmed by real-time PCR in the ccRCC cells after transfecting UBE2I shRNA. **(B, C)** The proliferation ability of A-498 cells **(B)** and 786-O cells **(C)** was measured by the CCK8 assay after transfecting shRNA. ***p < 0.001.

We knocked down UBE2I expression in 786-O and A-498 cells using shRNAs (Figure 9A). Results from CCK-8 experiments showed that cell proliferation was significantly reduced by UBE2I inhibition (Figures 9B,C).

These results indicated that UBE2I protein was highly expressed in ccRCC tissues, and a high-level nuclear translocation of UBE2I occurs in ccRCC. In addition, nuclear UBE2I expression could act as a potential novel promising diagnostic biomarker for ccRCC patients.

## Discussion

Genome-wide loss-of-function screen in a large number of tumor cell lines is an efficient method for identifying genes essential for the survival and proliferation of tumor cells. CRISPR/Cas9 technologies for gene editing have recently emerged as a powerful method for systematic loss-of-function screens, enabling precise genome-scale identification of genes essential to the tumor cell survival/proliferation. DepMap has developed a computational method (CERES) to assess the level of gene dependency based on CRISPR-Cas9 essentiality screens while considering the effect of copy-number–specific.

In the study, the dependence score was calculated by CERES and identified 735 genes essential to proliferation and survival of ccRCC cells from the DepMap website. Of the 735 genes, 485 DEGs were identified in ccRCC tumor tissues compared with normal tissues through the TCGA database. One module and 12 genes significantly related to ccRCC progression were identified through WGCNA, functional enrichment, and PPI. Enrichment analysis revealed that these 12 genes were enriched in the cell cycle pathway. Furthermore, we established a gene signature (UBE2I, NCAPG, NUP93, and TOP2A) screened from 12 genes, and this signature could divide patients into the low- and high-risk groups. Interestingly, the GSEA analysis indicated that the cell cycle pathway also ranked the first in the high-risk-score group, indicating its importance to the viability of ccRCC cells. Subsequently, the model was validated in the E-MTAB-1980-ccRCC and ICGC validation set. The result of tumor immunity analysis indicated that T-cell CD4 memory activated cells were positively correlated to the risk score. Previous studies showed that high infiltration of T-cell CD4 memory–activated cells predicted poor prognosis in ccRCC (Xu et al., 2021).

Generally, a dependency gene validated in cells did not directly represent its role in patients. First, their expression in adjacent normal and tumor tissues should be considered. Among these four genes, NCAPG, NUP93, and TOP2A were previously reported to be closely related to kidney disease, such as ccRCC. NCAPG encodes a subunit of the clusterin complex, which is responsible for the stability of chromosomes during meiosis and mitosis (Murphy and Sarge, 2008). A high level of NCAPG was significantly associated with unfavorable survival in various cancer types such as hepatocellular carcinoma, lung cancer, gastric cancer, ovarian cancer, breast cancer, cardia adenocarcinoma, and ccRCC (Liu et al., 2020; Peng et al., 2020; Xu et al., 2020; Wu et al., 2021; Liu et al., 2018; Zhang et al., 2020; Sun et al., 2020). In hepatocellular carcinoma, knockdown of NCAPG expression could not only reduce the viability of hepatocellular carcinoma cells, but also arrest the cells at the S phase of the cell cycle by regulating the expression of N-cadherin, E-cadherin, cleaved caspase-3, CDK2 (cyclin dependent–kinase 2), Bcl-2 (BCL2 apoptosis regulator), Bax (BCL2-associated X, apoptosis regulator), CCNA1 (cyclin A1), and HOXB9 (Homeobox B9) (Wang et al., 2019b). In cardia adenocarcinoma, NCAPG regulated the cell cycle and promoted cell proliferation by PI3K/AKT (AKT serine/threonine kinase) signaling pathway activation (Zhang et al., 2020). In lung adenocarcinoma, NCAPG promoted cell proliferation and migration through transforming growth factor β signaling pathway activation (Wu et al., 2021). In addition to the cell cycle, NCAPG may promote tumor development by regulating mismatch repair and cell senescence (Xiao et al., 2020).

NUP93 encodes a nucleoporin protein that localizes both to the basket of the pore and the nuclear entry of the central gated channel of the pore. It plays a central role in the assembly and maintenance of nuclear pore complex through anchoring nucleoporins to the nuclear pore complex (Grandi et al., 1997). Meanwhile, NUP93 is a target of caspase cysteine proteases that play an important role in programmed cell death by apoptosis. Diseases associated with NUP93 include nephrotic syndrome and genetic steroid-resistant nephrotic syndrome. NUP93 mutations can lead to steroid-resistant nephrotic syndrome (Braun et al., 2016). During renal development, NUP93 regulates the migration and proliferation of podocytes via SMAD4 (SMAD family member 4) signaling. Beyond that, NUP93 was found to be associated with tumor progression. The knockdown of NUP93 could inhibit the proliferation, invasion, and migration of cervical cancer cells (Ouyang et al., 2019). A high level of NUP93 was significantly associated with unfavorable survival in triple-negative breast cancer. NUP93 regulated the growth of breast cancer cells by modulating actin cytoskeleton remodeling and cell proliferation (Bersini et al., 2020).

TOP2A encodes a DNA topoisomerase, a protein that controls the DNA topologic states during transcription. It participates in several processes such as chromatid separation, chromosome condensation, and the relief of torsional stress that occurs during DNA replication and transcription. Aberrant expression of TOP2A has been identified in many tumors, including lung adenocarcinoma, hepatocellular carcinoma, adrenocortical carcinoma, neuroblastic tumors, prostate cancer, and breast cancer (de Resende et al., 2013; Jain et al., 2013; Moretti et al., 2013; Chen et al., 2016; Du et al., 2020; Zeng et al., 2020). TOP2A is overexpressed in ccRCC, and its overexpression promoted the proliferation and migration of ccRCC cells (Yuan et al., 2018; Luo et al., 2019; Zhang et al., 2019b). TOP2A could act as a predictor of response to epirubicin in the neoadjuvant treatment of breast cancer (Moretti et al., 2013).

UBE2I encodes an E2 SUMO-conjugating enzyme, also known as UBC9, which is required for SUMOylation to occur. SUMOylation is the covalent attachment of a small ubiquitin-like modifier (SUMO) protein to a lysine residue in a target protein, single UBE2I is central to the SUMOylation cascade (Rodriguez et al., 2019). UBE2I plays an important role in both cancer progression and chemotherapy resistance (Mo et al., 2004; Dünnebier et al., 2009; Ronen et al., 2009; Driscoll et al., 2010; Moschos et al., 2010; Qin et al., 2011). UBE2I was expressed at higher levels in lung cancer tissue compared with normal tissue, and upregulation of UBE2I expression promoted the migration and invasion of lung cancer cells (Li et al., 2013). UBE2I is the most highly expressed protein in protein extracts from melanoma infiltrated lymph nodes (Moschos et al., 2007). The up-regulation of UBE2I promoted the invasion and metastasis in breast cancer (Zhu et al., 2010). However, the role of UBE2I in ccRCC has not been explored previously.

As most of the substrates of SUMOylation are nuclear proteins including many cofactors or transcription factors, UBE2I can affect a variety of cellular pathways, such as cell proliferation, growth, and apoptosis (Baek, 2006). BRCA1 (BRCA1 DNA repair associated) is known as a cytoplasm–nuclear shuttling protein, and many tumor-associated mutations have altered the subcellular localization of BRCA1 protein (Bogdani et al., 2002). Previous study demonstrated that UBE2I may be required for nuclear import of BRCA1 (Qin et al., 2011). UBE2I has been considered to play a central role in SUMOylation. It has been shown previously that substrates need to be targeted to the nucleus for SUMOylation (Rodriguez et al., 2001; Wood et al., 2003). Therefore, UBE2I nuclear localization is expected to be critical for SUMOylation. Consistent with this, we observed that a high-level nuclear translocation of UBE2I occurs in ccRCC. The results indicated that SUMOylation may participate in ccRCC progression. However, whether it is a necessary condition for ccRCC still needs further research. In addition, nuclear UBE2I expression could act as a potential novel promising diagnostic biomarker for ccRCC patients. Therefore, exploring the transport of the UBE2I protein from the cytoplasm to the nucleus and blocking the translocation process of UBE2I may have potential therapeutic value with ccRCC patients.

## Conclusion

We combined functional genomic screening with gene expression to systematically study the genes that affect cell viability, and the cell cycle is an important pathway in this process. In addition, we have also established a risk score model consisting of four expression-driven dependency genes, including UBE2I, NCAPG, NUP93, and TOP2A. The prediction model has been further validated in ICGA and E-MTAB-1980 database. Finally, the IHC confirmed UBE2I accumulation in the nuclear of ccRCC tissues. Blocking the nuclear translocation of UBE2I may have potential therapeutic value with ccRCC patients.

## Data Availability

The datasets presented in this study can be found in online repositories. The names of the repository/repositories and accession number(s) can be found in the article/Supplementary Material.
