# Supplementary figures and images for: Identification of UBE2I as a Novel Biomarker in ccRCC Based on a Large-Scale CRISPR-Cas9 Screening Database and Immunohistochemistry
Source: Front Mol Biosci. 2022 Feb 8;9:813428. doi: 10.3389/fmolb.2022.813428 (PMC8861443; doi:10.3389/fmolb.2022.813428)

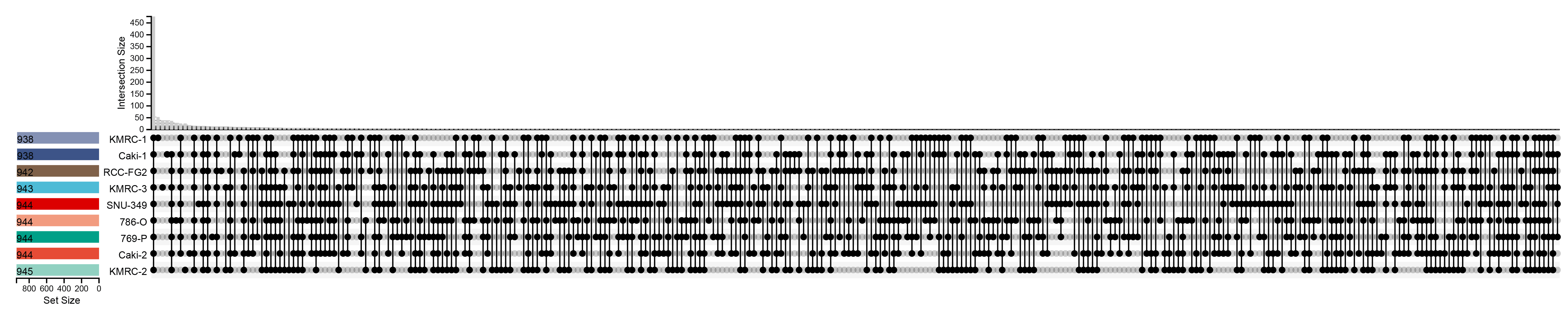

Supplement: Supplementary file 1 [file Image1.JPEG]
